# Time trends and geographical variation in prescribing of drugs for diabetes in England from 1998 to 2017

**DOI:** 10.1111/dom.13346

**Published:** 2018-06-05

**Authors:** Helen J. Curtis, John M. Dennis, Beverley M. Shields, Alex J. Walker, Seb Bacon, Andrew T. Hattersley, Angus G. Jones, Ben Goldacre

**Affiliations:** ^1^ Evidence‐Based Medicine DataLab, Nuffield Department of Primary Care Health Sciences University of Oxford Oxford UK; ^2^ Health Statistics Group, Institute of Health Research, University of Exeter Medical School Exeter UK; ^3^ Royal Devon and Exeter Hospital, Institute of Biomedical and Clinical Science, University of Exeter Medical School Exeter UK

**Keywords:** antidiabetic drug, cost‐effectiveness, glycaemic control, primary care, type 2 diabetes

## Abstract

**Aims:**

To measure the variation in prescribing of second‐line non‐insulin diabetes drugs.

**Materials and Methods:**

We evaluated time trends for the period 1998 to 2016, using England's publicly available prescribing datasets, and stratified these by the order in which they were prescribed to patients using the Clinical Practice Research Datalink. We calculated the proportion of each class of diabetes drug as a percentage of the total per year. We evaluated geographical variation in prescribing using general practice‐level data for the latest 12 months (to August 2017), with aggregation to Clinical Commissioning Groups. We calculated percentiles and ranges, and plotted maps.

**Results:**

Prescribing of therapy after metformin is changing rapidly. Dipeptidyl peptidase‐4 (DPP‐4) inhibitor use has increased markedly, with DPP‐4 inhibitors now the most common second‐line drug (43% prescriptions in 2016). The use of sodium‐glucose co‐transporter‐2 (SGLT‐2) inhibitors also increased rapidly (14% new second‐line, 27% new third‐line prescriptions in 2016). There was wide geographical variation in choice of therapies and average spend per patient. In contrast, metformin was consistently used as a first‐line treatment in accordance with guidelines.

**Conclusions:**

In England there is extensive geographical variation in the prescribing of diabetes drugs after metformin, and increasing use of higher‐cost DPP‐4 inhibitors and SGLT‐2 inhibitors compared with low‐cost sulphonylureas. Our findings strongly support the case for comparative effectiveness trials of current diabetes drugs.

## 1 INTRODUCTION

The prevalence of diabetes recorded across England in 2015 to 2016 was 6.55% of the population, or 3.03 million people.^1^ Good control of blood glucose in people with diabetes is important to reduce the risk of complications, and is measured primarily by maintenance of glycated haemoglobin (HbA1c) levels. Most people with diabetes are prescribed glucose‐lowering medication to achieve adequate glucose control.^2^ In 2016, antidiabetes drugs were identified as the section of National Health Service (NHS) prescribing with the greatest spend.^3^ In 2016 to 2017, 11.0% of England's total primary care net ingredient costs (NICs) were spent on diabetes, costing £984 m.^4^ While 54.9% of this was spent on insulin and diagnostic/monitoring items, the remaining £444 m went on the subset of “other antidiabetic drugs” (paragraph 6.1.2 of the British National Formulary [BNF]). These are drugs largely used to control type 2 diabetes, including metformin, sulphonylureas and several newer classes.

Metformin is recommended by the National Institute for Health and Care Excellence (NICE) as a first‐line treatment, but for many patients this is not sufficient to control the disease, and they are prescribed an additional, second‐line treatment.^2^ The optimal drug choice after metformin is unclear, with four different treatments recommended by NICE to form a dual therapy with metformin:^5^ sulphonylureas, pioglitazone (a thiazolidinedione [TZD]), dipeptidyl peptidase‐4 (DPP‐4) inhibitors and sodium‐glucose co‐transporter‐2 (SGLT‐2) inhibitors. The latter are recommended mainly if other therapies are contraindicated, but for the former three there is no particular order, and there is limited guidance on how they may be selected for different patients, except for some contraindications for pioglitazone. For patients requiring further intensification, triple therapy should comprise metformin and one of three possible combinations of two of the aforementioned drugs, or a fifth class, glucagon‐like peptide‐1 (GLP‐1) analogues, to be considered for obese patients.

Based on a sample of >400 000 people with type 2 diabetes on medication in 2013, 83.6% were receiving metformin, including 91.0% of patients receiving their first treatment.^2^ For patients requiring second‐line therapy after metformin, 61.7% received a sulphonylurea and 26.9% a DPP‐4; however, since then, SGLT‐2 inhibitors have become available and 2015 NICE guidelines markedly departed from previous guidance which recommended sulphonylureas as preferred therapy after metformin.

It is unclear how this situation is evolving and whether the different available medications are offered to patients in a consistent manner across the country. There is wide variation in cost between these treatment options, with metformin and sulphonylureas costing an average of ~£4 to £6 per item prescribed in 2015/2016, and SGLT‐2 and DPP‐4 inhibitors ~£40 per item.^1^

The aim of the present study was to determine variation in prescribing patterns and prescribing costs of antidiabetic treatments both geographically, across practices in England, and over time, by using three different datasets, summarized in Table S1 (Appendix S1).

## 2 METHODS

### 2.1 Data sources and preparation

We used 3 sources of data: the Clinical Practice Research Datalink (CPRD), a UK‐representative database of anonymized primary care electronic health records;^6^ annual Prescription Cost Analysis (PCA) data, aggregated nationally, covering 1998 to 2016; and monthly practice‐level prescribing data, from September 2010 to August 2017.

### 2.2 CPRD data

We extracted clinical and prescription records for 207 338 people with type 2 diabetes from the CPRD (download date January 19, 2017) who were prescribed a first‐ to fourth‐line oral diabetes drug, classified in the BNF 6.1.2 as “other antidiabetic drugs,” over the period 1998 to 2016 and had not previously been prescribed insulin. A detailed description of CRPD data ascertainment has been previously reported.^7^ Briefly, we positively identified patients with type 2 diabetes, largely on the basis of prescriptions rather than diagnostic medical codes because of known problems with coding errors^8^; however, we excluded patients with diagnostic codes for other forms of diabetes (eg, steroid‐induced, monogenic etc.) or polycystic ovary syndrome (which can be treated with metformin). To remove patients with type 1 diabetes, we excluded patients with an age at diagnosis of <35 years or those on insulin treatment within 12 months of diagnosis. We defined the date of diabetes diagnosis as the earliest of: first prescription for a non‐insulin diabetes therapy; first HbA1c result >47.5 mmol/mol (6.5%); or first diabetes diagnostic code. Ethics approval was granted by the CPRD Independent Scientific Advisory Committee (ISAC 13_177RA4R).

### 2.3 Annual PCA data

The annual PCA datasets contain one row for each treatment and dose, for all prescriptions issued in NHS primary care and dispensed in community settings in England, describing the number of prescriptions dispensed and the total cost. PCA data were processed as previously described.^9^, ^10^ Briefly, data for each year between 1998 and 2016 were obtained from NHS Digital or government archives and compiled. To correct for changes in drug names, spellings and classifications over time, each drug was assigned its full BNF code, chemical and product name from the current BNF. Drug names not matched exactly to a currently available product were assigned appropriate classifications via approximate matching. Data were normalized by converting the number of prescriptions and costs to relative figures per thousand population, using mid‐year populations for England.^11^ Number of items represents the number of times each drug was prescribed; costs are NICs, which represent the basic price of the medicine, that is, the price listed in the Drug Tariff or published by the manufacturer or supplier. NICs may be subject to further charges and/or discounts. Costs were also corrected for inflation using the consumer price index compared with 2016.^12^

### 2.4 Practice‐level data

The monthly prescribing datasets published by NHS Digital contain one row for each treatment and dose, in each prescribing organization in NHS primary care in England, describing the number of prescriptions issued and the total cost. We limited this to organizations with setting code “4”– general practices, according to the NHS Digital dataset of practice characteristics,^13^ to exclude all other organizations, such as prisons and out‐of‐hours services. Practices with a current status of “closed” or “dormant” were also excluded from the latest 12 months' analysis. Each practice in England belongs to one of 207 Clinical Commissioning Groups (CCGs) which are responsible for commissioning healthcare services in their local area, so we aggregated these data for CCG‐level analyses. We used number of items, which represents the number of times each drug was prescribed, and actual costs, which are the full cost to the NHS including NICs and any further charges and discounts.

### 2.5 Prevalence data

Estimates of percentage national prevalence of type 2 diabetes for 2000 to 2013 were obtained from an analysis of practices in The Health Improvement Network (THIN) database,^2^ extrapolated to cover 1998 to 2016 using a straight‐line estimation in Excel (R^2^ = 0.9965). We calculated items prescribed/cost per person with diabetes for England by dividing prescribing figures per 1000 population by the prevalence rate per 1000. CCG and practice‐level prevalence figures were obtained from the Quality Outcomes Framework (QOF)^14^ for the financial year 2016 to 2017 (April to March) and including all types of diabetes. To estimate the number of patients with diabetes per CCG, accounting for missing practice registrations, we multiplied the adult prevalence rate from the QOF by the population (aged ≥15 years) of each CCG, using each CCG's latest practice membership, at August 2017. We then calculated rates of items prescribed per person with diabetes by dividing prescribing figures by the number of people registered with diabetes in the corresponding population.

### 2.6 Extraction and classification of diabetes drug data

In the CPRD we categorized drug prescriptions using BNF codes and Medcode keyword searches of “product name” and “drug substance name.” The CPRD includes full prescription records but no data on drug dispensation. New drug prescriptions (and their corresponding start dates) were defined as the first‐ever prescription of a drug in each class for each patient, even if only prescribed once. Patients were considered to have stopped a drug if there was a gap in prescribing of that drug of at least 6 months.^7^ We defined first‐, second‐, third‐ or fourth‐line prescription categories based on the order of new drug prescriptions for individual patients. Every time a patient started a new drug we assigned this to the next line of therapy, regardless of whether their concomitant therapy changed at a similar time point.

In the PCA and practice‐level datasets we extracted the prescribing data for paragraph 6.1.2, “other antidiabetic drugs.” Drugs were each assigned to the appropriate class (metformin, sulphonylureas, TZDs, gliptins/DPP‐4 inhibitors, GLP‐1 analogues and SGLT‐2 inhibitors) based on their chemical name (Table S2, Appendix S1). In CPRD, we assigned combination drug prescriptions containing metformin and one other to both constituent classes; in the PCA and practice‐level datasets these combination drugs were counted only as the non‐metformin drug (eg, metformin hydrochloride/rosiglitazone was assigned to the class of rosiglitazone, ie, TZDs). In all datasets, drugs containing a mixture of any other two classes were counted as “other.”

### 2.7 Analysis

We calculated prescribing rates per class of drug by dividing the number of items prescribed by the total number of antidiabetic items (BNF 6.1.2) prescribed. Trend charts from PCA data were produced in Excel by summing items or cost per patient with type 2 diabetes over each class per year. In the CPRD, we calculated the proportion of new prescriptions of each drug for each calendar year and line of therapy as the total number of new prescriptions of the drug/total number of new prescriptions. CPRD data extraction and analysis was conducted in stata v14.0, and trends charts were produced using Excel. Deciles of practice‐level prescribing trends across all practices were calculated for each available month and plotted as time trend charts using Python. After limiting to and aggregating the latest available 12 months, summary tables of CCG and practice prescribing were produced in Python.

### 2.8 Maps

Maps of antidiabetic items prescribed by all CCGs in England for a single month snapshot were created using http://openprescribing.net/analyse, by selecting all the chemicals within each class as numerator and BNF Paragraph 6.1.2 as denominator. The results were converted to percentages (from per 1000). The data source for http://openprescribing.net is the monthly practice‐level prescribing dataset described above. It includes all practices with status “4” (standard general practices), but does not exclude closed and dormant practices. The map of spend per patient across CCGs was produced using Tableau Open software.

### 2.9 Data and code

PCA and practice‐level data were extracted using SQL in Google BigQuery. The links to each map for CCG prescribing on http://openprescribing.net are provided in the Supporting Information. The Tableau workbook mapping the costs per patient is available online at https://public.tableau.com/views/Diabetesmap/Dashboard1?:embed=y&:display_count=yes. Practice‐level data were analysed and charts produced using Python *scipy.stats*, *matplotlib.pyplot* and *seaborn* modules. Complete codes are provided in the Supporting Information and PCA data extract in FigShare.^15^

## 3 RESULTS

### 3.1 Data sources and preparation

From the CPRD we included data on 392 764 new first‐ to fourth‐line antidiabetic drug prescriptions for 207 338 unique patients in the period 1998 to 2016. Of these, 48% (191 550) were for a first‐line drug, 31% (124 673) for a second‐line, 14% (57 679) for a third‐line and 5% (18 862) for a fourth‐line drug. Metformin was the most common newly initiated drug prescribed (48% of total new prescriptions), followed by sulphonylureas (27%), TZDs (10%), DPP‐4 inhibitors (10%), GLP‐1 analogues (2%) and SGLT‐2 inhibitors (2%). All PCA data were extracted successfully. From the practice‐level data for the latest 12 months, 7261 practices belonging to all 207 current CCGs were included, after excluding 342 with a status of closed (68) or dormant (274). In total this covered 48 million registered patients (aged ≥15 years), of which ~3.1 million were registered as having diabetes. For decile calculations for the period 2010 to 2017, in total 8155 practices were included.

### 3.2 Prescription rates of second‐, third‐ and fourth‐line diabetes therapies

**Figure 1:**
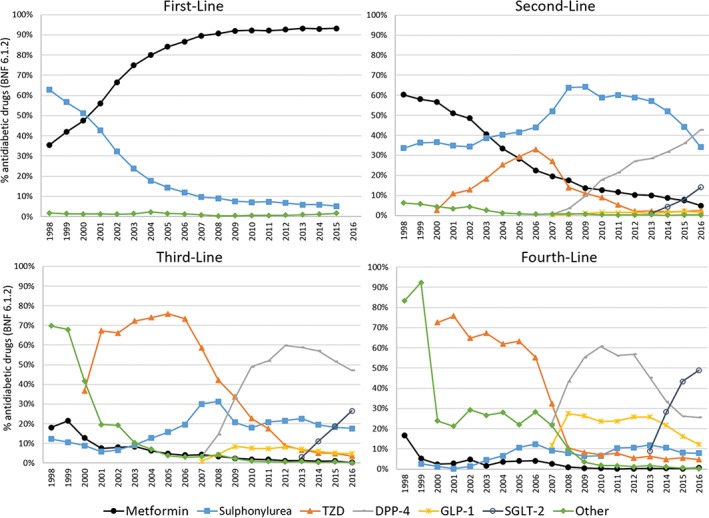
Time trends in antidiabetic medication prescribed across the United Kingdom, 1998 to 2016, separated by line of therapy (First‐Line, Second‐Line, Third‐Line and Fourth‐Line, ie, the order in which additional drugs were prescribed to each patient), based on Clinical Practice Research Datalink data. The prescriptions for each class of antidiabetic drug each year are given as a percentage of all antidiabetic prescriptions (British National Formulary [BNF] 6.1.2). In the First‐Line chart, all classes other than metformin and sulphonylurea are grouped into “Other”. DPP‐4, dipeptidyl peptidase‐4; GLP‐1, glucagon‐like peptide‐1; SGLT‐2, sodium‐glucose co‐transporter‐2; TZD, thiazolidinedione

The CPRD data show that metformin has been favoured as first‐line treatment since 2001 (Figure 1), with a rate of >90% for the past 10 years. Sulphonylureas were the most common second‐line therapy, prescribed in 40% to 64% of cases from 2004 to 2015. In 2016, the most common second‐line therapy became DPP‐4 inhibitors, with second‐line sulphonylurea use rapidly declining to 34%. Metformin use in second‐line therapy has declined over time (from 60% to 5%) as most patients began to receive it as a first‐line treatment. In third‐line therapies, DPP‐4 inhibitors have remained the most common since 2010, but declined slightly since reaching a peak of 60% in 2012. The use of SGLT‐2 inhibitors as second‐, third‐ and fourth‐line therapy is rising rapidly. It has been the most common fourth‐line therapy since 2015, prescribed to almost half of patients requiring a fourth drug. GLP‐1 analogues appear to be largely reserved for third‐ or fourth‐line therapy only.

### 3.3 Long term trends in prescribing for diabetes in England

**Figure 2:**
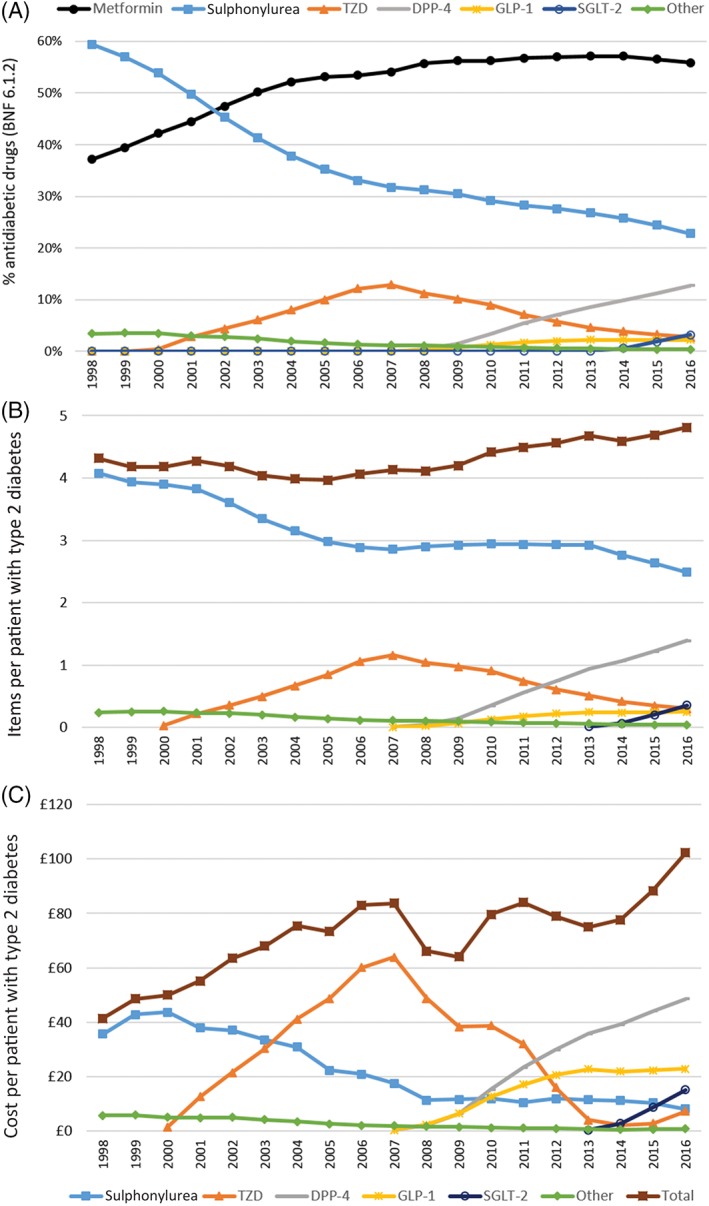
Time trends in antidiabetic medications dispensed in English primary care in Prescription Cost Analysis data, 1998 to 2016. A, Proportion of each class of drug dispensed in England each year, taking items prescribed of each as a percentage of all antidiabetic items prescriptions (British National Formulary [BNF] 6.1.2). B, Number of items and C, inflation‐corrected cost of each class of (and total) non‐metformin diabetes drug dispensed in England per person with type 2 diabetes. DPP‐4, dipeptidyl peptidase‐4; GLP‐1, glucagon‐like peptide‐1; SGLT‐2, sodium‐glucose co‐transporter‐2; TZD, thiazolidinedione

Figure 2 shows the national prescribing of non‐insulin glucose‐lowering therapy in the PCA data for the period between 1998 and 2016. Trends in overall prescribing (Figure 2A) are similar to those found in the CPRD. The total number of non‐metformin items dispensed relative to the number of patients with diabetes was relatively stable, increasing only modestly since 2008 (4.1‐4.8 items per year, 17%). Costs per patient, however, have risen by 55% since 2008, from £66 to £102.

From 2014 to 2016, a 32% increase in cost per patient (£78 to £102) was accompanied by only a 5% increase in items dispensed (4.6‐4.8). This rise corresponds with increased usage of DPP‐4 and SGLT‐2 inhibitors. An increase in the price of TZDs, caused by a shortage of generic pioglitazone^16^ and subsequent price volatility, also contributed to the rise.

Despite remaining the most widely used second‐line therapy, sulphonylureas contribute less to total cost per patient than most of the other classes because of their generic availability (Figure 2B,C). Recently, the use of sulphonylureas has been declining, apparently in favour of the newer therapies. Spend on TZDs has dropped substantially since its peak in 2007; this is attributable to both the decline in their usage and expiry of the patent. The spend on DPP‐4 inhibitors, SGLT‐2 inhibitors and GLP‐1 analogues is increasing in line with their usage. Despite the slow uptake of GLP‐1 analogues, they represent the second highest cost burden: in 2016, four times as many DPP‐4 inhibitors as GLP‐1 analogues were prescribed, but the expenditure only differed by a factor close to 2. National trends in prescribing of individual agents within each class are shown in Figure S1 (Appendix S1).

### 3.4 National variation in prescribing by CCGs

**Figure 3:**
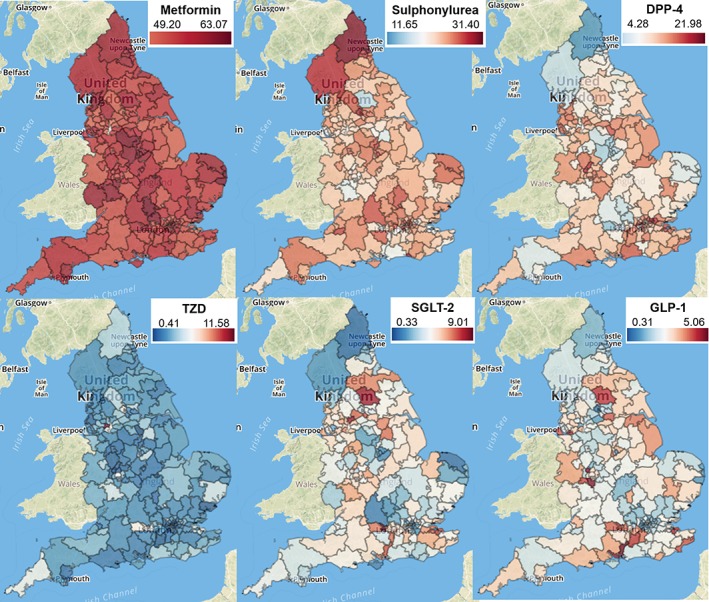
Geographical variation in prescribing of antidiabetic drugs by all Clinical Commissioning Groups in England, May 2017. For each class of drug (Metformin, Sulphonylurea, DPP‐4, TZD, SGLT‐2 and GLP‐1), numbers represent number of items prescribed as a percentage of all antidiabetic drugs prescribed (British National Formulary 6.1.2). Updated versions of each map may be accessed at http://openprescribing.net using links provided in Table S4 (Appendix S1). DPP‐4, dipeptidyl peptidase‐4; GLP‐1, glucagon‐like peptide‐1; SGLT‐2, sodium‐glucose co‐transporter‐2; TZD, thiazolidinedione

We investigated how the level of prescribing of each antidiabetic drug class varied across England, summarizing the proportions prescribed by each CCG over a 12‐month period (Table 1) and mapping them geographically for a single‐month snapshot (Figure 3). There was relatively low variation in metformin items as a proportion of all antidiabetic drugs (55.6% ± 2.9%; Table 1 and Figure 3), but more marked differences in the other available therapies favoured in each region. In the TZD class, the mean level of prescribing across CCGs was 2.5% ± 1.4% (Table 1), but one CCG consistently prescribed more (12% in May 2017; Figure 3).

**Table 1:** Volumes and cost of antidiabetic drugs prescribed across England's Clinical Commisioning Groups over a 12‐month period, September 2016 to August 2017

	Mean	SD	Median	Lower quartile	Upper quartile	IQR	Kurtosis
Metformin, %	55.6	2.9	55.2	53.6	57.6	4.0	8.0
Sulphonylurea, %	21.6	3.5	21.6	19.2	24.0	4.9	5.0
DPP‐4, %	13.5	3.3	13.9	11.3	16.0	4.7	2.7
TZD, %	2.5	1.4	2.2	1.5	3.0	1.5	8.7
SGLT‐2, %	4.1	1.6	4.2	2.9	5.2	2.3	10.2
GLP‐1, %	2.4	0.9	2.4	1.8	2.9	1.1	46.2
Non‐metformin, non‐sulphonylurea (%)	22.8	4.7	23.6	20.0	26.2	6.2	
Items per patient with diabetes	11.6	2.0	11.4	10.4	12.8	2.5	
Cost per patient with diabetes	£130	£25	£131	£114	£148	£35	

Abbreviations: DPP‐4, dipeptidyl peptidase‐4; GLP‐1, glucagon‐like peptide‐1; IQR, interquartile range; SGLT‐2, sodium‐glucose co‐transporter‐2; TZD, thiazolidinedione.

Percentages represent the proportion of items for each drug class out of all anti‐diabetic items prescribed (British National Formulary paragraph 6.1.2). The total number of items and cost of antidiabetic prescribing per patient are also given. SD and kurtosis for percentage measures are included as metrics of variation between regions.

The spend on antidiabetic drugs per patient with diabetes over the latest 12 months ranged from £60 to £200 across CCGs (Figure S2, Appendix S1). Lower cost per patient generally corresponded to lower rates of prescribing of non‐metformin, non‐sulphonylurea classes (Figure S2, Appendix S1), but the variation in total prescribing level per patient (11.6 ± 2.0) may also be a contributor (Table 1). The total spend was £414 m over this period; however, if every CCG could have prescribed at the lowest decile cost per patient (£95 per patient) this would represent a saving of £113 m, which is more than a quarter of the total costs for this area of prescribing.

### 3.5 National variation in prescribing at practice level

**Figure 4:**
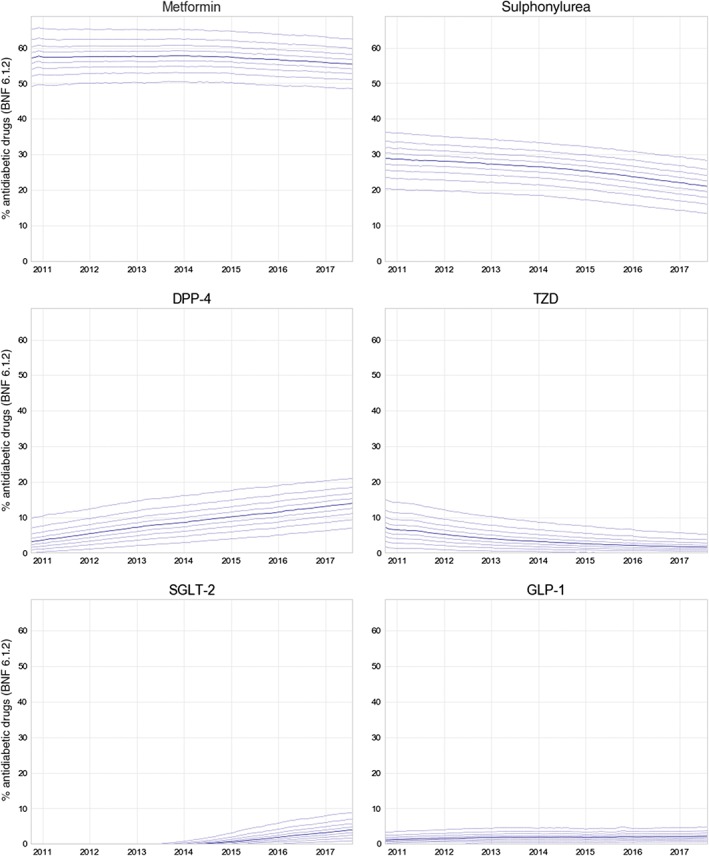
Decile charts summarizing the proportion of each drug class of all antidiabetic items prescribed (British National Formulary [BNF] paragraph 6.1.2) across England's general practices, between October 2010 and August 2017. For each class of drug (Metformin, Sulphonylurea, DPP‐4, TZD, SGLT‐2 and GLP‐1), solid lines represent the median, dashed lines are 10th to 90th percentiles. DPP‐4, dipeptidyl peptidase‐4; GLP‐1, glucagon‐like peptide‐1; SGLT‐2, sodium‐glucose co‐transporter‐2; TZD, thiazolidinedione

As expected, variation was greater across practices than when aggregated to CCGs (Table S3, Appendix S1); the proportion of metformin prescribed extended to a range of ~40% to 70%, but with interquartile range restricted to 52% to 59%. This range of variation has remained roughly constant since 2010 (Figure 4). Almost all practices prescribe at least some sulphonylureas and DPP‐4 inhibitors, with interquartile ranges of 17.9% to 25.5% and 9.8% to 16.8% respectively. The remaining three classes are commonly prescribed in small proportions, with medians close to 0, and for 75% of practices they each make up <6% of antidiabetic medications (Table S3, Appendix S1). The rise of the SGLT‐2 inhibitor class is highly variable across practices (Figure 4).

## 4 DISCUSSION

We have assessed variation in NHS primary care prescribing of diabetes treatments both geographically, across CCGs and practices in England, and over time. There is wide regional variation in choice of second‐line therapy, reflecting the absence of clear evidence or guidelines to inform treatment choice. The more specific guidelines concerning metformin are well adhered to overall, with relatively little variation in metformin use across regions and over time. Recent prescribing increasingly favours the newer more expensive treatments, leading to a rapid increase in cost of prescribing over recent years.

A key strength of the present analysis is that it uses three datasets, with overlapping strengths and weaknesses. The CPRD contains data on individual patients, which permits selection of only those with type 2 diabetes and investigation of the order in which medicines were prescribed for individual patients. However, the CPRD covers only a small subset of all prescribing, and does not permit exploration of individual institutions' prescribing at the level of identifiable CCGs and practices, while the PCA and practice‐level datasets cover the complete data for all primary care prescribing in England, not a sample, down to the level of all practices and CCGs, and PCA data cover all national‐level prescribing back to 1998.

While prescriptions issued by a hospital clinic or private practice, or dispensed in hospital, are not included in our data, almost all prescriptions for glucose‐lowering agents in the United Kingdom are issued through a general practice. Even agents started on the recommendation of a hospital or community endocrinologist or other specialist will almost always be prescribed through general practice and captured in our data, with the exception being inpatient and emergency prescribing. Our data are therefore an accurate representation of UK prescribing practice. Using number of items prescribed in PCA and practice‐level data does not distinguish between different lengths of courses being prescribed (eg, 1 month's supply vs 3 months'), therefore drugs prescribed in shorter courses may amount to a greater total number of items. Converting into average daily quantities or defined daily doses would be an improvement, but a comprehensive dataset to render this calculation for all medications does not exist, and antidiabetes drugs may be given in different dosages. Using quantity instead would help to overcome this problem, but does not allow fair comparison between drugs given in different dosing regimens, or between items prescribed in different units of measurement such as liquids (mL) and tablets. In the CPRD, individual prescribing data were available, including dose and prescription frequency, and the finding of consistent results across CPRD and PCA data is a strength of the present study. Although the data do not indicate adherence to therapy, the focus of our study was on prescribing choices.

Correcting for diabetes prevalence allowed us to investigate variation in prescribing independently from the increasing number of people living with the condition. National type 2 diabetes prevalence data for 1998 to 2016 were extrapolated from estimates from THIN practices.^2^ The advantage of these data is that the coverage of the sample is comprehensive, including secondary care data. The figures reported in the National Diabetes Audit (NDA) were lower, but prevalence in the NDA was lower than predicted from epidemiological studies because some patients were not registered as having diabetes by practices.^17^ The QOF figures were ~0.7 to 0.8 percentage points lower than the figures used, probably because the QOF includes all types of diabetes and excludes those aged <17 years, the age group with lowest prevalence; however, like the NDA, QOF data also depend on practice registrations which may be incomplete. QOF figures were the best available source of practice and CCG‐level data on prevalence.

Similarly to the United Kingdom, type 2 diabetes guidelines from the American College of Physicians, the European Association for the Study of Diabetes and the American Diabetes Association all leave the choice of therapy after metformin largely to the practitioner^18^, ^19^, ^20^; therefore, our key findings, that the use of therapy after metformin is changing dramatically and that there is geographical variation in drug prescribing in England, are likely to be generalizable to other countries.

We found that metformin has been favoured as first‐line treatment since 2001, reflecting guidance and previous reports.^2^ The rise in metformin use has been attributed to the publication of several UK‐based studies on the efficacy of metformin from 1998, which had a high level of media publicity, with the later NICE guidance having no clear additional effect.^21^ DPP‐4 inhibitors are thought to have a similar efficacy to sulphonylureas but cause fewer side effects.^22^ The increasing use of SGLT‐2 inhibitors may relate to a favourable side effect profile in previous trials, which includes weight loss, oral administration (in comparison with injected GLP‐1 inhibitors, the only other class associated with clinically significant weight loss), and (for empagliflozin), positive cardiovascular outcomes.^23^ Empagliflozin was, however, only the third most common SGLT‐2 inhibitor prescribed in England in 2015 and 2016. If newer antidiabetic drugs succeed in reducing serious side effects and complications, additional spend on these drugs may lead to reduced spend on other related healthcare costs, and may also improve patient experience. Indeed, the spend on antidiabetic drugs in 2010 was estimated to make up just 6.1% of the total cost of drugs and care for people with type 2 diabetes in the United Kingdom.^24^

The relationship between type 2 diabetes prescribing levels and HbA1c control across practices participating in the NDA (>50%) has recently been studied.^25^ Greater HbA1c control was correlated with higher levels of metformin and DPP‐4 inhibitor prescribing, lower prescribing of sulphonylureas, and lower overall spend on diabetes medication per patient (including estimated quantities of blood testing strips and insulin used for people with type 2 diabetes). Greater achievement on non‐pharmaceutical targets was also correlated with better HbA1c control.^25^ Such variability in care and outcomes has led to the initiation of trials studying interventions targeted at primary care practitioners, including their prescribing behaviour.^26^, ^27^

Previous work has shown that responses to unclear guidelines can be variable. When a common antipsychotic drug had its licence severely restricted but no specific advice was given on which alternative drug should be prescribed, in Scotland chlorpromazine was the most common replacement, whereas in England it was a combination of chlorpromazine and two newer drugs,^28^ but regional variation within England was not studied. Similarly, the removal of the licence for co‐proxamol was followed by an increase in several other analgesics.^29^ In addition, while safety concerns around prescribing tend to be acted upon quickly, evidence‐based guidelines have less impact, even when the prescribing advice is clear, suggesting that dissemination could be improved.^30^, ^31^

We found unexplained variation in choice of non‐metformin treatment, in the context of absence of clear advice in guidelines and current evidence. Aside from clinicians' personal choices, there may be a variety of external influences, including local policy, price changes, marketing, financial arrangements with drug companies, media reports, access to educational material, and drug safety alerts. The present findings raise various prospects and opportunities in diabetes research. Firstly, they suggest that a randomized trial of choice of second‐line medication would be clinically useful to resolve outstanding uncertainty on the best treatment for an extremely common clinical presentation. There is no such study ongoing in the United Kingdom, which seems a remarkable oversight, given that diabetes is the single biggest cost area for prescribing in NHS England. The one such study ongoing in the United States, GRADE, does not include SGLT‐2 inhibitor therapy.^32^ Secondly, the findings suggest that a pragmatic low‐cost cluster randomized trial, randomizing practices or CCGs to a prescribing policy that prefers a particular second‐line treatment, would be justifiable on the grounds of costs and ethics, as there is already existing unexplained variation.^33^

Thirdly, in the absence of guidance on which second‐line treatment is best, and with guidance only suggesting that the lowest cost options within each class are preferred, we found extensive variation in prescribing costs between CCGs. The total spend was £414 m over 12 months, but with a potential saving of £113 m if all CCGs had prescribed at the same per‐patient cost as the most efficient decile of CCGs; however, a full cost‐effectiveness analysis would require consideration of differences in side effects and cardiovascular outcomes across the different drug classes as well as consideration of non‐medicinal treatments. Our http://openprescribing.net project is an openly accessible data service which highlights prescribing variation in primary care, and allows practices and commissioners to monitor their own prescribing behaviour for key prescribing measures and any chemical of interest, using statistical process control techniques to send alerts automatically to practices when they deviate from national changes in behaviour (including on diabetes prescribing). We have previously argued that greater investment in disseminating evidence, auditing its implementation, and using variation in practice to target clinicians for educational interventions may all prove to be cost‐effective mechanisms to ensure that health services use treatments effectively and cost‐effectively.

In conclusion, with a lack of good evidence to guide choice of second‐line treatment for diabetes, we found evidence of extensive variation in choice of drug, and prescription volumes for new treatments rising as they appear on the market, in the absence of good comparative effectiveness data.

## Supporting information


**Appendix 1.** Additional Tables and Figures
**Table S1.** Comparison of key features of each dataset used in our study, and the list of figures and tables for which each dataset is used.
**Table S2.** Assignment of BNF drug names to diabetes drug classes.
**Table S3.** Proportion of each drug class prescribed across England's practices, September 2016 to August 2017.
**Table S4.** Links to updated maps as shown in Figure 3.
**Figure S1.** Prescribing trends for individual chemicals within each class of anti‐diabetes drug in England, 1998 to 2016.
**Figure S2.** Map of total spend on anti‐diabetic drugs per patient and prescribing level of newer anti‐diabetic drugs, by CCG.
**Appendix 2.** SQL and Python Codes for data extraction and analysis.
