# Supplementary material for: Time trends and geographical variation in prescribing of drugs for diabetes in England from 1998 to 2017
Source: Diabetes Obes Metab. 2018 Jun 5;20(9):2159–68. doi: 10.1111/dom.13346 (PMC6099452; doi:10.1111/dom.13346)
Supplement: Supplementary file 1 — Appendix 1. Additional Tables and Figures Table S1. Comparison of key features of each dataset used in our study, and the list of figures and tables for which each dataset is used. Table S2. Assignment of BNF drug names to diabetes drug classes. Table S3. Proportion of each drug class prescribed across England's practices, September 2016 to August 2017. Table S4. Links to updated maps as shown in Figure 3. Figure S1. Prescribing trends for individual chemicals within each class of anti‐diabetes drug in England, 1998 to 2016. Figure S2. Map of total spend on anti‐diabetic drugs per patient and prescribing level of newer anti‐diabetic drugs, by CCG. Appendix 2. SQL and Python Codes for data extraction and analysis. [file DOM-20-2159-s001.pdf]

Time Trends and Geographical Variation in Prescribing of Drugs for Diabetes in England 1998-2017

Supplementary Information

Contents

Appendix 1: Additional Tables and Figures

Table S1:	Comparison of key features of each dataset used in our study, and the list of Figures and Tables for which each dataset is used.
Table S2:	Assignment of BNF drug names to diabetes drug classes.
Table S3:	Proportion of each drug class prescribed across England's practices, September 2016-August 2017.
Table S4:	Links to updated maps as shown in Figure 3.
Figure S1:	Prescribing trends for individual chemicals within each class of anti-diabetes drug in England, 1998-2016.
Figure S2:	Map of total spend on anti-diabetic drugs per patient and prescribing level of newer anti-diabetic drugs, by CCG.

Appendix 2: SQL and Python Codes for data extraction and analysis

APPENDIX 1

Table S1. *Comparison of key features of each dataset used in our study, and the list of Figures and Tables for which each dataset is used.*

	CPRD	PCA	Practice-level
Patient-level†	✓		
Long term (from 1998)	✓	✓	
Freely available		✓	✓
National coverage		✓	✓
Identifiable organisations			✓
Monthly data	✓		✓
Figures	*1*	*2, S1*	*3, 4, S2*
Tables			*1, S3, S4*

†Patient-level data allows selection of patients by diagnosis, and determination of the sequence in which drugs are prescribed to individual patients.

Table S2. *Assignment of BNF drug names to diabetes drug classes. Other drugs were assigned to class "Other".*

Chemical code	Chemical name	Class
0601023A0	Acarbose	Alpha-glucosidase
0601023AU	Ins Degludec/Liraglutide	Combination
0601023AV	Saxagliptin/Dapagliflozin	Combination
0601023AA	Vildagliptin	DPP-4
0601023AC	Saxagliptin	DPP-4
0601023AD	Metformin Hydrochloride/Sitagliptin	DPP-4
0601023AE	Linagliptin	DPP-4
0601023AF	Linagliptin/Metformin	DPP-4
0601023AH	Saxagliptin/Metformin	DPP-4
0601023AJ	Alogliptin/Metformin	DPP-4
0601023AK	Alogliptin	DPP-4
0601023X0	Sitagliptin	DPP-4
0601023Z0	Metformin Hydrochloride/Vildagliptin	DPP-4
0601023AB	Liraglutide	GLP-1
0601023AI	Lixisenatide	GLP-1
0601023AQ	Dulaglutide	GLP-1
0601023Y0	Exenatide	GLP-1
0601022B0	Metformin Hydrochloride	Metformin
0601023R0	Repaglinide	Prandial glucose regulator
0601023U0	Nateglinide	Prandial glucose regulator
0601023AG	Dapagliflozin	SGLT-2

0601023AL	Dapagliflozin/Metformin	SGLT-2
0601023AM	Canagliflozin	SGLT-2
0601023AN	Empagliflozin	SGLT-2
0601023AP	Canagliflozin/Metformin	SGLT-2
0601023AR	Empagliflozin/Metformin	SGLT-2
0601021A0	Glimepiride	Sulfonylurea
0601021E0	Chlorpropamide	Sulfonylurea
0601021H0	Glibenclamide	Sulfonylurea
0601021M0	Gliclazide	Sulfonylurea
0601021P0	Glipizide	Sulfonylurea
0601021R0	Gliquidone	Sulfonylurea
0601021V0	Tolazamide	Sulfonylurea
0601021X0	Tolbutamide	Sulfonylurea
0601023B0	Pioglitazone Hydrochloride	TZD
0601023S0	Rosiglitazone	TZD
0601023V0	Metformin Hydrochloride/Rosiglitazone	TZD
0601023W0	Metformin Hydrochloride/Pioglitazone	TZD

Table S3. *Proportion of each drug class prescribed across England's practices, September 2016-August 2017.*

	Mean	Std Dev	Median	Lower Quartile	Upper Quartile	IQR	Kurtosis
**Metformin (%)**	55.8	5.6	55.7	52.5	59.1	6.6	8.0
**Sulfonylurea (%)**	21.7	5.7	21.8	17.9	25.5	7.6	5.0
**DPP-4 (%)**	13.4	5.3	13.3	9.8	16.8	7.0	2.7
**TZD (%)**	2.4	2.5	1.8	0.7	3.3	2.6	8.7
**SGLT-2 (%)**	4.0	3.0	3.4	1.8	5.5	3.7	10.2
**GLP-1 (%)**	2.4	1.7	2.1	1.2	3.2	1.9	46.2
**Non_Met_Non_SU (%)**	22.6	7.2	22.4	17.7	27.1	9.4	3.3
**Total Anti-Diabetic Items**	5,142	3,448	4,416	2,770	6,675	3,905	
**Total Anti-Diabetic Costs**	£56,967	£38,929	£48,568	£29,758	£75,269	£45,510	
**Items per diabetic**	12.3	3.7	12.0	9.6	14.4	4.8	
**Cost per diabetic**	£136	£44	£132	£106	£160	£54	

Percentages represent the proportion of items for each drug out of all anti-diabetic items prescribed (BNF paragraph 6.1.2). Standard deviation (and kurtosis for percentage measures) are included as metrics of variation in between regions. Items and Cost per Diabetic figures exclude 95 practices with no prevalence recorded in QOF. Non-Met Non-SU = Non-metformin, non-sulfonylurea.

Table S4. *Links to updated maps as shown in Figure 3. Maps show relative volumes of each class of anti-diabetic drug prescribed, per 1000 items of all anti-diabetic items (BNF 6.1.2), for each CCG in England.*

Metformin	https://openprescribing.net/analyse/#org=CCG&numIds=0601022B0&denomIds=6.1.2&selectedTab=map
Sulfonylurea	https://openprescribing.net/analyse/#org=CCG&numIds=0601021H0,0601021M0,0601021P0,0601021A0,0601021X0,0601021E0&denomIds=6.1.2&selectedTab=map
All others	https://openprescribing.net/analyse/#org=CCG&numIds=0601023AK,0601023AJ,0601023AE,0601023AF,0601023AD,0601023Z0,0601023AC,0601023AH,0601023X0,0601023AA,0601023B0,0601023S0,0601023V0,0601023W0,0601023AM,0601023AP,0601023AG,0601023AL,0601023AN,0601023AR,0601023Y0,0601023AB,0601023AI,0601023AQ&denomIds=6.1.2&selectedTab=map
DPP-4	https://openprescribing.net/analyse/#org=CCG&numIds=0601023AK,0601023AJ,0601023AE,0601023AF,0601023AD,0601023Z0,0601023AC,0601023AH,0601023X0,0601023AA&denomIds=6.1.2&selectedTab=map
TZD	https://openprescribing.net/analyse/#org=CCG&numIds=0601023B0,0601023S0,0601023V0,0601023W0&denomIds=6.1.2&selectedTab=map
SGLT-2	https://openprescribing.net/analyse/#org=CCG&numIds=0601023AM,0601023AP,0601023AG,0601023AL,0601023AN,0601023AR&denomIds=6.1.2&selectedTab=map
GLP-1	https://openprescribing.net/analyse/#org=CCG&numIds=0601023Y0,0601023AB,0601023AI,0601023AQ&denomIds=6.1.2&selectedTab=map
DPP-4, SGLT-2 and GLP-1	https://openprescribing.net/analyse/#org=CCG&numIds=0601023AK,0601023AJ,0601023AE,0601023AF,0601023AD,0601023Z0,0601023AC,0601023AH,0601023X0,0601023AA,0601023AM,0601023AP,0601023AG,0601023AL,0601023AN,0601023AR,0601023Y0,0601023AB,0601023AI,0601023AQ&denomIds=6.1.2&selectedTab=map

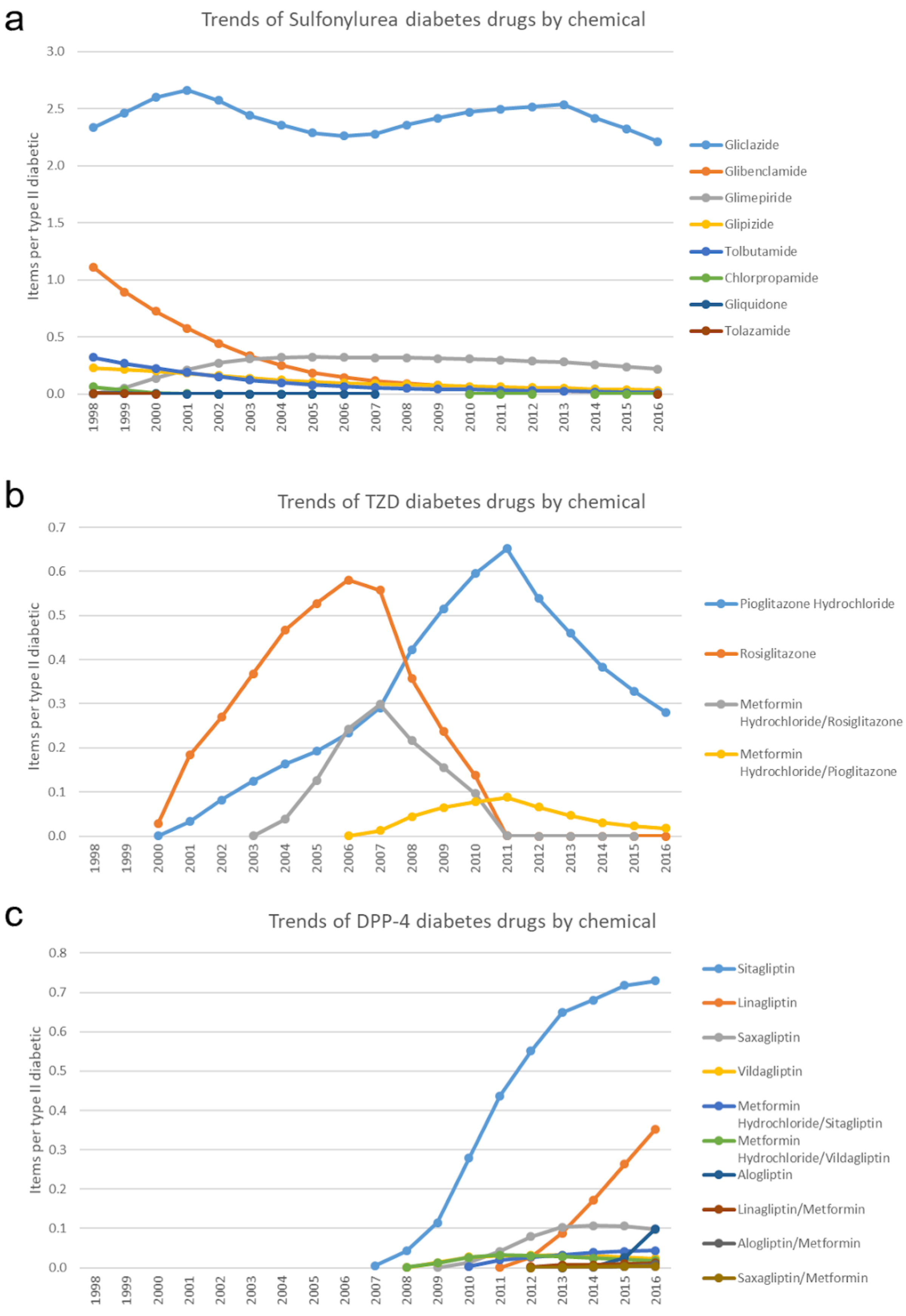
a
Trends of Sulfonylurea diabetes drugs by chemical
Items per type II diabetic
Gliclazide
Glibenclamide
Glimepiride
Glipizide
Tolbutamide
Chlorpropamide
Gliquidone
Tolazamide
b
Trends of TZD diabetes drugs by chemical
Items per type II diabetic
Pioglitazone Hydrochloride
Rosiglitazone
Metformin Hydrochloride/Rosiglitazone
Metformin Hydrochloride/Pioglitazone
c
Trends of DPP-4 diabetes drugs by chemical
Items per type II diabetic
Sitagliptin
Linagliptin
Saxagliptin
Vildagliptin
Metformin Hydrochloride/Sitagliptin
Metformin Hydrochloride/Vildagliptin
Alogliptin
Linagliptin/Metformin
Alogliptin/Metformin
Saxagliptin/Metformin

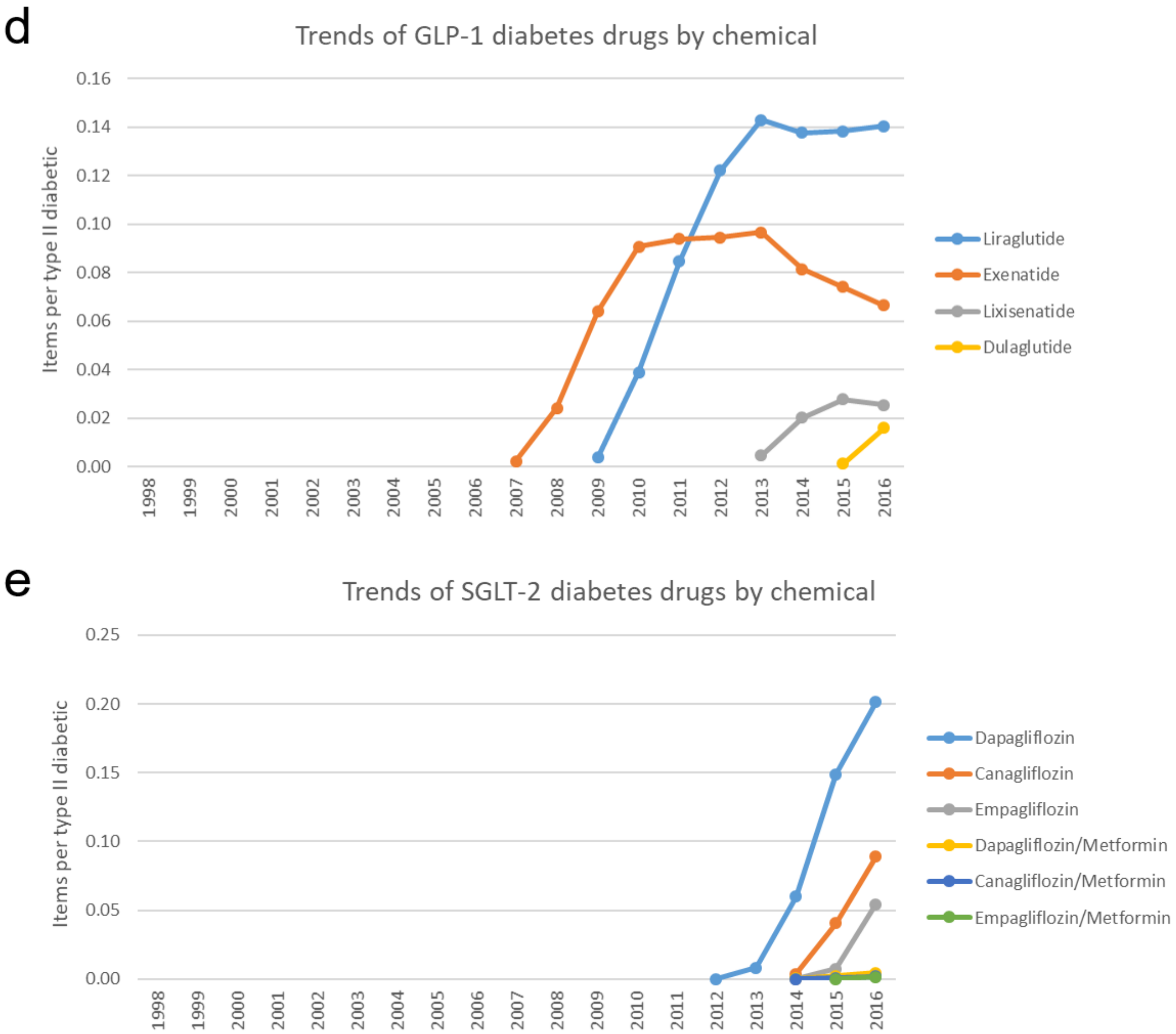

Figure S1. *Prescribing trends for individual chemicals within each class of anti-diabetes drug in England, 1998-2016. Number of items dispensed per type II diabetic according to PCA data, within each class of diabetes drug: (a) Sulfonylurea, (b) TZD, (c) DPP-4, (d) GLP-1, (e) SGLT-2.*

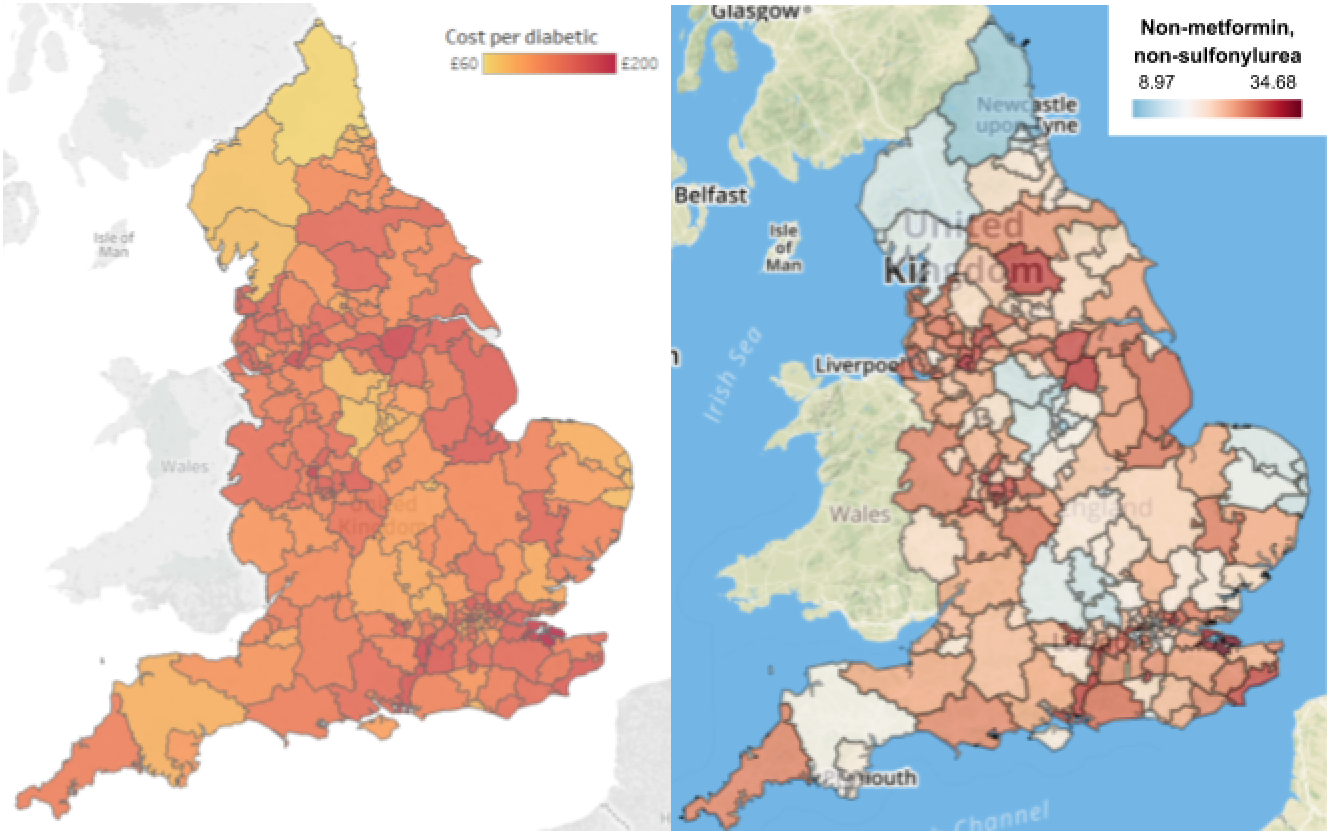

Figure S2. *Map of total spend on anti-diabetic drugs per patient and prescribing level of newer anti-diabetic drugs, by CCG. A: Total spend on anti-diabetic drugs, Sept 2016-Aug 2017, corrected for number of patients with diabetes. B. Percentage of non-metformin, non-sulfonylurea anti-diabetic items prescribed of all anti-diabetic drugs (BNF paragraph 6.1.2), May 2017, from OpenPrescribing.net.*

APPENDIX 2 - SQL and Python Codes for data extraction and analysis

1. SQL code
    a. Extraction of all practice-level data on anti-diabetic drugs.

```
SELECT * FROM ebmdatalab.hscic.prescribing
WHERE bnf_code like '060102%'
-- save as table ebmdatalab.helen.diabetes_prescribing
```

    b. Extraction of all diabetes prescribing data from normalised PCA dataset

```
SELECT a.*, COALESCE(l.class, "Other") AS class
FROM ebmdatalab.helen.trends_from_pca_final_2016 a
LEFT JOIN ebmdatalab.helen.diabetes_drug_class_lookup2 l on
      l.chemical_name = a.chemical_current
WHERE section_current = "Drugs Used In Diabetes"
      AND (para_code_current = '2' OR l.class IS NOT NULL)
ORDER BY year
```

2. Python scripts for analysis of practice-level data:
    a. Summary tables and savings calculations:
    https://gist.github.com/HelenCEBM/ebf64c09c39e6c1d706eaf08cc2cd422
    b. Decile charts:
    https://gist.github.com/HelenCEBM/b1f4068b1619c8dbb4578a3300c644ce
